# Granulocyte macrophage colony-stimulating factor predicts postoperative recurrence of clear-cell renal cell carcinoma

**DOI:** 10.18632/oncotarget.8235

**Published:** 2016-03-21

**Authors:** Yuan Chang, Le Xu, Lin Zhou, Qiang Fu, Zheng Liu, Yuanfeng Yang, Zongming Lin, Jiejie Xu

**Affiliations:** ^1^ Department of Urology, Zhongshan Hospital, Fudan University, Shanghai, China; ^2^ Department of Biochemistry and Molecular Biology, School of Basic Medical Sciences, Fudan University, Shanghai, China; ^3^ Department of Urology, Ruijin Hospital, School of Medicine, Shanghai Jiaotong University, Shanghai, China

**Keywords:** clear-cell renal cell carcinoma, granulocyte macrophage colony-stimulating factor, prognostic biomarker, recurrence-free survival, nomogram

## Abstract

**Background:**

Granulocyte macrophage colony-stimulating factor (GM-CSF) is currently widely used as an adjuvant in cancer immunotherapy. However, recent studies have shown that GM-CSF can impair anti-tumor immune responses. Thus the role of GM-CSF in clear-cell renal cell carcinoma (ccRCC) remains unraveled. Our present study aims to investigate the prognostic significance of intratumoral GM-CSF in patients with clinically localized ccRCC.

**Results:**

A high intratumoral GM-CSF expression was significantly associated with lymph node metastases (*P* = 0.009), high TNM stage (*P* = 0.031), high Fuhrman grade (*P* < 0.001), presence of tumor necrosis (*P* = 0.005), and high Leibovich scores (*P* < 0.001). In addition, the prognostic significance of intratumoral GM-CSF expression was restricted to patients with Leibovich intermediate/high-risk (*P* = 0.001). Furthermore, a high intratumoral GM-CSF expression was demonstrated as an independent prognostic factor of reduced RFS (*P* = 0.018). Incorporation of the intratumoral GM-CSF expression into a prognostic model including TNM stage, Fuhrman grade, tumor necrosis and lymphovascular invasion generated a nomogram, which predicted accurately 3- and 5-year survival for ccRCC patients.

**Materials and Methods:**

This study comprised 233 clinically localized (T1-3N0-1M0) ccRCC patients undergoing nephrectomy in 2008 at a single centre. Intratumoral GM-CSF expression was assessed by immunohistochemical staining and its associations with clinicopathologic features and recurrence-free survival (RFS) were evaluated.

**Conclusions:**

The intratumoral GM-CSF expression, as a potentially independent prognostic biomarker for recurrence, might improve conventional clinical and pathologic analysis to refine outcome prediction for clinically localized ccRCC patients after surgery.

## INTRODUCTION

Clear-cell renal cell carcinoma (ccRCC) is the most common type of kidney cancer and accounts for 60% to 70% of all renal tumors. Despite advances in diagnosis, especially for improved abdominal imaging, more ccRCC patients can be diagnosed at an early stage [1]. Currently, surgery remains the most effective treatment for clinically localized RCC, however, nearly 30% of patients undergoing curative nephrectomy progress to metastasis or experience local recurrence during follow-up, which leads to a poor prognosis [2]. Because of the highly variable natural history of RCC, outcomes for patients with similar clinicopathologic features differ significantly.

By far, TNM staging system [3] and Fuhrman grading system [4] remain the most commonly used prognostic systems for RCC patient outcome. Several integrated clinical and pathologic prognostic models have been established to identify patients who have higher risk of disease progression after surgery, such as the Mayo Clinic stage, size, grade and necrosis (SSIGN) score [5] to predict cancer-specific survival for ccRCC and the Leibovich score [6] to predict recurrence-free survival (RFS) for clinically localized ccRCC. Nevertheless, accurate prediction of individual RCC biology is still difficult. It is expected that a combination of specific molecular biomarkers into traditional clinicopathologic characteristics will allow better prediction of prognosis [7].

Granulocyte macrophage colony-stimulating factor (GM-CSF) is known as a potent hematopoietic growth factor for granulocyte and macrophage expansion and is considered to play a critical role in anti-tumor responses by dendritic cells (DCs) maturation and T cell proliferation and activation [8]. However, clinical trials in metastatic RCC (mRCC) using GM-CSF as single-agent or combined with either interleukine-2 (IL-2) or interferon-α (IFN-α) have failed to show anti-tumor effect [9–11]. Although RCC vaccination studies using GM-CSF as immune adjuvant have shown promising results, it is not clear whether this cytokine is indispensable for the clinical improvement [12, 13]. Meanwhile, human RCC cells can directly secrete significant amount of GM-CSF [14], and recent studies have revealed an immunosuppressive effect of GM-CSF by expansion of myeloid-derived suppressor cells (MDSCs) and stimulation of FoxP3^+^ regulatory T cells (Tregs) in blood and tumor microenvironment, which leads to concerns about potential detrimental effects of this cytokine [15–17]. Therefore, the role of GM-CSF in RCC remains elusive.

In this study, we sought to investigate associations between intratumoral GM-CSF expression with clinicopathologic features and prognostic value in clinically localized ccRCC. GM-CSF expression was assessed by immunohistochemistry (IHC) in ccRCC specimens. Moreover, a nomogram combined intratumoral GM-CSF expression with TNM stage, Fuhrman grade, tumor necrosis and lymphovascular invasion was established to predict 3- and 5-year RFS for clinically localized ccRCC patients after nephrectomy.

## RESULTS

### Intratumoral GM-CSF expression and its associations with patient clinicopathologic features

**Figure 1:**
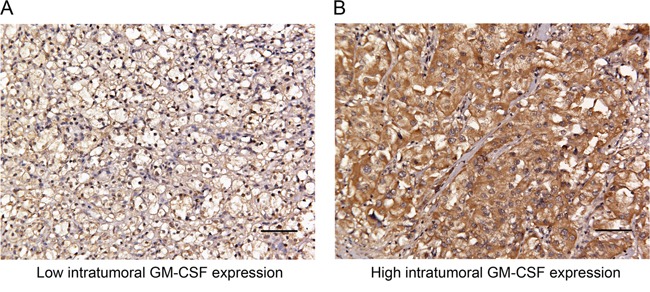
Representative photographs of intratumoral GM-CSF expression by immunostaining in clinically localized ccRCC Low intratumoral GM-CSF expression **A.,** high intratumoral GM-CSF expression **B.** Scale bar = 100μm. Original magnification ×200.

Intratumoral GM-CSF expression was evaluated by IHC analysis in 233 ccRCC specimens. GM-CSF positive staining was predominantly located in the cytoplasm (Figure 1A and 1B). According to the cutoff value derived from the IRS score, 40.0% (93/233) and 60.1% (140/233) were scored as high and low intratumoral GM-CSF expression, respectively. The detailed clinicopathologic characteristics and their associations with intratumoral GM-CSF expression were summarized in Table 1. Median follow-up was 68 months (interquartile range, 41 to 71 months), and at last follow-up there were 56 (25.8%) patients confirmed with tumor recurrence at a median of 30 months following surgery (interquartile range, 12 to 51 months). The 3- and 5-year RFS rates were 86.6% and 76.0%, respectively. High GM-CSF expression was significantly associated with lymph node metastases (*P* = 0.009), high TNM stage (*P* = 0.031), high Fuhrman grade (*P* < 0.001), presence of tumor necrosis (*P* = 0.005), and high Leibovich scores (*P* < 0.001) (Table 1).

**Table 1:** Patient characteristics and associations with intratumoral GM-CSF expression

Characteristic	Total patients (*n* = 233)		GM-CSF		
	No.	%	Low (*n* = 140)	High (*n* = 93)	*P*
**Age, year**					0.453*
Median	56.0		56.0	55.0	
IQR	48.0-62.0		48.0-63.8	48.0-61.0	
**Gender**					0.343
Male	170	73.0	99	71	
Female	63	27.0	41	22	
**Pathologic T stage**					0.065
T1	145	62.2	92	53	
T2	24	10.3	17	7	
T3	64	27.5	31	33	
**Pathologic N stage**					0.009
N0	228	97.9	140	88	
N1	5	2.1	0	5	
**Fuhrman grade**					<0.001
1	41	17.6	33	8	
2	96	41.2	67	29	
3	56	24.0	29	27	
4	40	17.2	11	29	
**Tumor necrosis**					0.005
Absent	182	78.1	118	64	
Present	51	21.9	22	29	
**LVI**					0.208
Absent	166	71.2	104	62	
Present	67	28.8	36	31	
**Leibovich score**					<0.001
0-2	105	45.1	75	30	
3-5	95	40.8	54	41	
≥6	33	14.2	11	22	

GM-CSF = granulocyte macrophage colony-stimulating factor; IQR = interquartile range; LVI = lymphovascular invasion.

* Wilcoxon rank-sum test; chi-square test for all the other analyses.

### Associations of intratumoral GM-CSF expression with RFS

**Figure 2:**
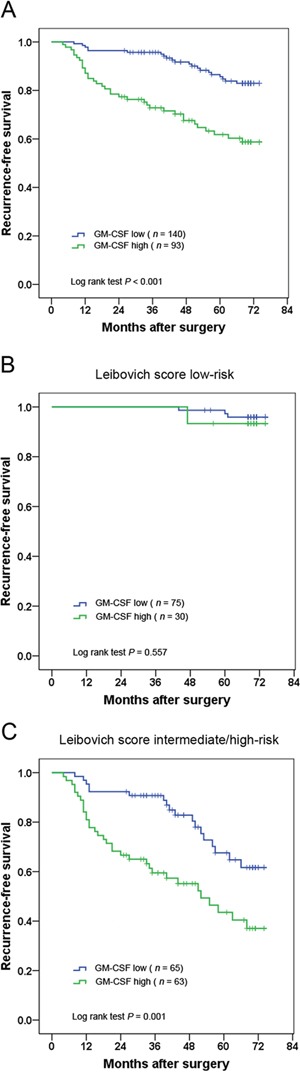
Kaplan–Meier analysis for recurrence-free survival (RFS) of clinically localized ccRCC patients according to intratumoral GM-CSF expression Kaplan–Meier analysis for RFS in 233 ccRCC patients **A.,** 105 ccRCC patients with Leibovich score low-risk **B.,** 128 ccRCC patients with Leibovich score intermediate/high-risk **C.**

The association of intratumoral GM-CSF expression with RFS is illustrated by Kaplan-Meier curves. Patients with high GM-CSF expression had a significantly poorer RFS (*P* < 0.001) (Figure 2A). The 5-year RFS rates for high and low GM-CSF group were 62.4% and 85.0%, respectively. To investigate further the effect of intratumoral GM-CSF expression in stratifying patients with different Leibovich scores, we grouped the Leibovich 0–2 scores, 3–5 scores and ≥ 6 scores as low-risk, intermediate-risk and high-risk, respectively. By Kaplan–Meier analysis, the prognostic value of intratumoral GM-CSF expression was observed to be restricted to patients with Leibovich score intermediate/high-risk (*P* = 0.001) (Figure 2B and 2C).

Furthermore, univariate and multivariate analyses were performed to investigate whether the GM-CSF expression was an independent prognostic predictor of RFS. In the univariate analysis, the GM-CSF expression had prognostic significance for RFS (*P* < 0.001). The multivariate analysis demonstrated that the GM-CSF expression (*P* = 0.018), TNM stage (*P* < 0.001), Fuhrman grade (*P* < 0.001), tumor necrosis (*P* < 0.001) and lymphovascular invasion (*P* < 0.001) were independent prognostic factors of RFS in clinically localized ccRCC (Table 2).

**Table 2:** Univariate and multivariate Cox proportional hazards regression analysis for recurrence-free survival

Variables	Univariate			Multivariate		
	HR	95% CI	*P*	HR	95% CI	*P*
**Age†**	1.023	0.999-1.047	0.064			
**Sex**			0.737			
Female	Reference					
Male	1.109	0.606-2.031				
**TNM stage**			<0.001			<0.001
I	Reference			Reference		
II	3.869	1.701-8.802		5.408	2.158-13.554	
III	4.525	2.532-8.088		6.582	3.352-12.926	
**Fuhrman grade**			<0.001			<0.001
1+2	Reference			Reference		
3	3.225	1.622-6.410		2.304	1.102-4.819	
4	12.735	6.602-24.568		8.525	3.729-19.493	
**Tumour necrosis**			<0.001			<0.001
Absent	Reference			Reference		
Present	5.597	3.287-9.532		4.117	2.211-7.664	
**LVI**			<0.001			<0.001
Absent	Reference			Reference		
Present	3.507	2.067-5.950		3.114	1.735-5.588	
**GM-CSF**			<0.001			0.018
Low	Reference			Reference		
High	3.181	1.850-5.472		2.013	1.130-3.584	

GM-CSF = granulocyte macrophage colony-stimulating factor; HR = hazard ratio; CI = confidence interval; LVI = lymphovascular invasion.

† Analyzed as a continuous variable.

### Predictive nomogram for RFS

**Figure 3:**
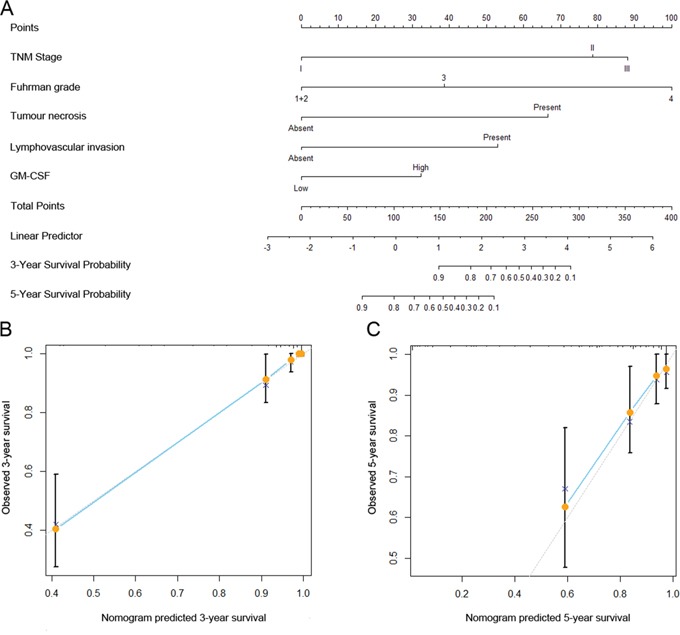
Nomogram for predicting 3- and 5-year recurrence-free survival (RFS) of clinically localized ccRCC patients after surgery Nomogram for predicting 3- and 5-year RFS of ccRCC patients after surgery **A.** Calibration plot of the nomogram for 3-year **B.** and 5-year survival **C.** The dashed line represents the performance of an ideal nomogram. The blue line indicates the performance of the proposed nomogram. Orange circles are sub-cohorts of the data set; X is the bootstrapped corrected estimate of nomogram with 200 resamples. Vertical bars represent 95% CI. It seems that the nomogram predicts accurately 3- and 5-year RFS.

To provide a quantitative method for better outcome prediction, we constructed a nomogram that integrated the proven independent prognostic factors consisting of TNM stage, Fuhrman grade, tumor necrosis, lymphovascular invasion and intratumoral GM-CSF expression (Figure 3A). In this nomogram, a higher total point indicates a worse RFS. For internal validation, calibration plots of the nomogram predicting 3- and 5-year survival performed well with the ideal model (Figure 3B and 3C). The *C*-index of the multivariate prognostic model based on TNM stage, Fuhrman grade, tumor necrosis and lymphovascular invasion was 0.867 and improved to 0.879 when the intratumoral GM-CSF expression was incorporated, which showed a better predictive ability of RFS than Leibovich scores (*C*-index 0.850).

## DISCUSSION

In the current study, we investigated the correlations of intratumoral GM-CSF expression with clinicopathologic characteristics and prognosis of 233 clinically localized ccRCC patients after surgical treatment. We demonstrated that a high GM-CSF expression was an independent and adverse predictor of RFS in multivariate analysis. In addition, the prognostic value of GM-CSF expression was restricted to patients with Leibovich score intermediate/high-risk. Together with TNM stage, Fuhrman grade, tumor necrosis and lymphovascular invasion, intratumoral GM-CSF expression was integrated in a prognostic nomogram that predicted RFS with an accuracy of 0.879, which showed a more accurate prediction than Leibovich score. Thus, our study serves as a proof of principal that intratumoral GM-CSF expression can provide important prognostic information that augments conventional clinicopathologic analysis. It is hoped that additional, prospective and clinical validation studies will be conducted to confirm our findings.

GM-CSF is secreted as a single chain glycoprotein stimulating bone-marrow precursor cells differentiation into both granulocytes and macrophage colonies [18]. Although the exact immunostimulatory function of GM-CSF is unclear, it is believed that it exerts its role by enhancing local recruitment and maturation of DCs and subsequently increasing antigen presentation. Thus, GM-CSF is currently widely used as an adjuvant in cancer immunotherapy. However, in a phase II trial, Rini et al found that GM-CSF administrated subcutaneously has little activity against mRCC [10]. Several clinical trials showed that the combination of GM-CSF, IL-2 and IFN-α had only limited efficacy in a selection group of mRCC patients without distinguishing between any possible synergistic or additive impact of any of these agents [9, 19].

Resent work has established the ability of GM-CSF to impair the immunization. Myeloid-derived suppressor cells (MDSCs), a heterogeneous population of myeloid progenitors, represent an immune cell subset which can suppress immune function and support tumor proliferation and metastasis [20]. Tregs also hold an important role in impeding immune surveillance against cancer and hampering the development of effective anti-tumor immunity [21]. In a previous report, increased presence of intratumoral Tregs was significantly associated with worse cancer-specific survival of RCC [22]. Serafini et al unraveled that the dual face of GM-CSF which is able to either enhance or impair anti-tumor immunity in a dose-dependent manner: a high-dose of GM-CSF may prevent immune responses by recruiting MDSCs [17]. This was further supported by the research of Filipazzi et al, who identified the presence of MDSCs in peripheral blood of melanoma patients treated with subcutaneous administration of recombinant GM-CSF [15]. In addition, Jinushi et al found that GM-CSF could induce milk fat globule EGF 8 expression on antigen-presenting cells, resulting in the efficient phagocytosis of apoptotic cells, and the maintenance of Tregs in the periphery [16]. Furthermore, Gerharz et al observed that human RCCs were able to release abundant GM-CSF to modulate the tumor-directed immune responses, but the associations of tumor progression with the differences in the amount of GM-CSF between different RCCs remain obscure [14]. Based on the above findings, concerns about the opposite effect of GM-CSF applied in clinical setting have been raised.

Parmiani et al reviewed plenty of previous reported cancer vaccination trials in which GM-CSF was used as adjuvant and found that GM-CSF could improve the vaccine-induced immune activity when administrated at relatively low-dose whereas an immunosuppressive effect was observed at relatively high-dose [23]. In consistent with the findings of Parmiani et al, we demonstrated that a high intratumoral GM-CSF expression was an independent predictor of diminished RFS for clinically localized ccRCC patients. Furthermore, we found that a high intratumoral GM-CSF expression was more likely to have aggressive tumor biological phenotypes including lymph node metastases, high TNM stage, high Fuhrman grade, presence of tumor necrosis, and high Leibovich scores. Thus, our results, as well as other related findings, imply that administration of GM-CSF even at a low-dose to ccRCC patients whose tumor tissues produce increased expression of GM-CSF may possibly worsen their clinical conditions. Since GM-CSF is a double-edged sword that exerts a central role in mediating immune homeostasis, caution should be marked in the clinical use of GM-CSF.

Although challenges exist, it is possible to solve these problems by developing suitable agents to counteract regulatory mechanisms that oppose successful immunotherapy and looking for predictive biomarkers that can improve our understanding of detailed regulatory mechanisms and predict an individual response to therapy. Given these considerations, Walter et al conducted clinical trials with administration of a single dose of cyclophosphamide prior to inoculation of the RCC vaccine composed of tumor peptides and GM-CSF in an effort to attenuate Treg responses and achieved promising results [13]. To date, the profound molecular roles of GM-CSF signaling in RCC remain far from being fully elucidated and need further investigation.

In tradition, outcome prediction in RCC patients is based on clinical and pathological factors such as TNM stage and Fuhrman grade, however, the natural history of RCC is complex. In the recent decade, molecular biomarkers of RCC are starting to offer additional means to predict tumor behavior and thereby improve patient outcome [7]. By incorporating the intratumoral GM-CSF expression into TNM stages, Fuhrman grade, tumor necrosis and lymphovascular invasion, a nomogram was constructed and performed well in internal validation. When assessing RFS, a higher predictive accuracy of the nomogram can be observed compared with that of Leibovich score. Moreover, we found that the prognostic value of intratumoral GM-CSF expression was predominately pronounced in patients with Leibovich score intermediate/high-risk, suggesting that GM-CSF could provide additional prognostic information as a complement to conventional clinical and pathological characteristics. These findings may facilitate clinicians to better select patients for participating in clinical trials of adjuvant therapy and customizing postoperative follow-up.

The major limitations of our study are retrospective nature and no external validation. Thus, a multicenter, prospective study is needed to validate these results in a larger population in the future. In addition, because this is a retrospective study, blood samples from patients are not available. The associations between serum-based GM-CSF levels and patient outcome are not evaluated and merit further investigation. Finally, although 2 tissue cores from the same tumor allowed the IHC staining to be verified, substantial intratumoral heterogeneity of RCC may impair the precise molecular analysis in this study [24].

In conclusion, the present study indicates that intratumoral GM-CSF expression may serve as an independent prognostic factor of RFS and should be incorporated into conventional clinical and pathological factors to refine outcome prediction of clinically localized ccRCC patients after surgery.

## MATERIALS AND METHODS

### Patients

This retrospective study included 233 patients with clinically localized (T1-3N0-1M0) ccRCC who underwent nephrectomy in our institute during the year of 2008. The exclusion criteria were as follows: non-clear cell RCC confirmed histopathologically, prior or concurrent distant metastasis, a history of previous anti-cancer therapies and other malignancies, bilateral renal cancer, perioperative mortality and tumor tissues unavailable. For each patient, the following clinical and pathologic information was gathered: age at surgery, gender, TNM stage [3], Fuhrman grade [4], tumor necrosis and lymphovascular invasion. Histopathologic review on each of the tumor specimens was performed by a single pathologist to confirm reported pathologic findings. Tumor necrosis was defined as the presence of microscopic coagulative necrosis. Lymphovascular invasion was defined as the presence of tumor cells within an endothelium-lined space without underlying muscular walls. The presence of nodal metastases was defined according to pathologic findings. The absence of distant metastases was defined according to radiographic examinations. The Leibovich score was applied to classify patients into three risk levels: 0-2, 3-5, and ≥6 scores based on pathologic T and N stage, tumor size, Furhman grade and necrosis [6].

Patients with clinically localized RCC were treated with radical or partial nephrectomy. Patients were followed up postoperatively with physical examination, laboratory studies, chest imaging, and abdominal ultrasound or computed tomography every 6 months for the first 2 years and annually thereafter. Follow-up was terminated in March 2014. Recurrence-free survival (RFS) was defined as time from surgery to recurrence or death or censored at the last follow-up date. This study was approved by Zhongshan hospital's Ethics Committee, and informed consent was obtained from each patient.

### Tissue microarray and immunochemistry

Tissue microarray (TMA) construction and immunohistochemistry protocol were described previously [25]. Briefly, duplicate cores in 1mm diameter were taken from two representative areas of each tumor tissue to construct TMA slides. The primary antibody against human GM-CSF-ab167552-(dilution1:100; Abcam, Cambridge, MA, USA) was applied in the procedure. All the cases were stained at once. A semiquantitative immunoreactivity scoring (IRS) was used for the evaluation of immuostaining by two independent pathologists blind to patient information. Staining intensity was scored (0, no staining; 1, weak; 2, moderate; 3, strong) and the percentage of positive tumor cells was scored (0% to 100%). The final score for each case was recorded by multiplying the score of staining intensity and the percentage of positive tumor cells, which ranges from 0 to 300. We selected the optimum cutoff score (160) for the expression of GM-CSF using X-tile software version3.6.1 (Yale University School of Medicine. New Haven. CT. USA) based on the association with patients’ RFS (Supplementary Figure S1).

### Statistical analyses

Analysis was performed with SPSS 21.0 (IBM Corporation, Armonk, NY, USA) and R software version 3.0.2 and the “rms” package (R Foundation for Statistical Computing, Vienna, Austria). Pearson χ^2^ test or Fisher's exact test was used to compare categorical variables, and continuous variables were analyzed by Wilcoxon rank-sum test. The Kaplan-Meier method with log-rank test was used to compare survival curves. The Cox proportional hazards regression model was applied to perform univariate and multivariate analyses and those variables that achieved statistical significance in the univariate analysis were entered into the multivariable analysis. Furthermore, a nomogram was created by R software using “rms” package. Calibration plots were generated to examine the performance characteristics of the predictive nomogram. The Harrell's Concordance index (*C*-index) was used to quantify the predictive accuracy [26], which ranges from 0.5 (no predictive power) to 1 (perfect prediction). All statistical tests were two-sided and performed at a significance level of 0.05.

## SUPPLEMENTARY FIGURE


