# Supplementary material for: Granulocyte macrophage colony-stimulating factor predicts postoperative recurrence of clear-cell renal cell carcinoma
Source: Oncotarget. 2016 Mar 21;7(17):24527–36. doi: 10.18632/oncotarget.8235 (PMC5029719; doi:10.18632/oncotarget.8235)
Supplement: Supplementary file 1 [file oncotarget-07-24527-s001.pdf]

SUPPLEMENTARY FIGURE

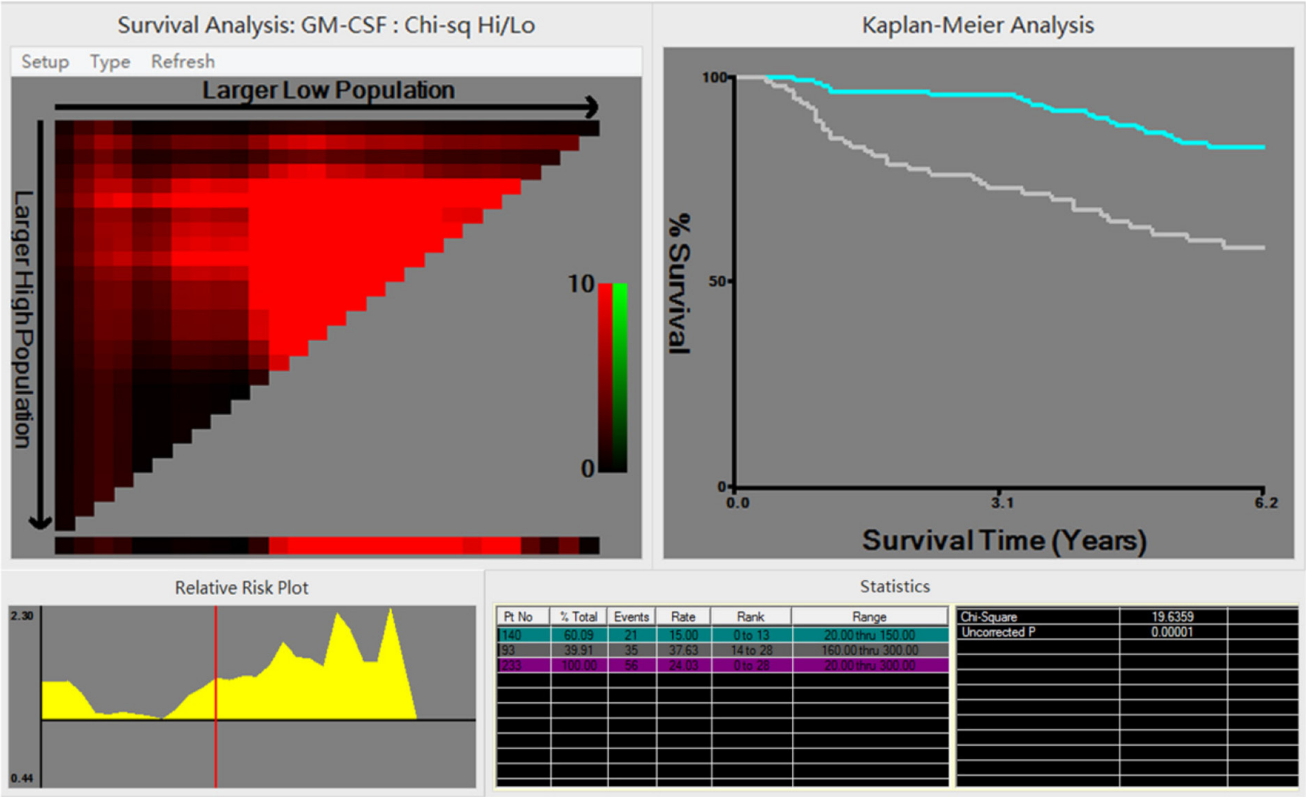

Supplementary Figure S1: The optimal cutoff value for the intratumoral GM-CSF expression was selected by X-tile 3.6.1 software (Yale University School of Medicine, New Haven, CT, USA).
